# Highly efficient optogenetic cell ablation in *C. elegans* using membrane-targeted miniSOG

**DOI:** 10.1038/srep21271

**Published:** 2016-02-10

**Authors:** Suhong Xu, Andrew D. Chisholm

**Affiliations:** 1 Division of Biological Sciences, Section of Cell and Developmental Biology, University of California, San Diego, 9500 Gilman Drive, La Jolla, CA 92093.

## Abstract

The genetically encoded photosensitizer miniSOG (mini Singlet Oxygen Generator) can be used to kill cells in *C. elegans*. miniSOG generates the reactive oxygen species (ROS) singlet oxygen after illumination with blue light. Illumination of neurons expressing miniSOG targeted to the outer mitochondrial membrane (mito-miniSOG) causes neuronal death. To enhance miniSOG’s efficiency as an ablation tool in multiple cell types we tested alternative targeting signals. We find that membrane targeted miniSOG allows highly efficient cell killing. When combined with a point mutation that increases miniSOG’s ROS generation, membrane targeted miniSOG can ablate neurons in less than one tenth the time of mito-miniSOG. We extend the miniSOG ablation technique to non-neuronal tissues, revealing an essential role for the epidermis in locomotion. These improvements expand the utility and throughput of optogenetic cell ablation in *C. elegans*.

Cell ablation is a classic approach to understanding cell and tissue function in development or neural function *in vivo*. In *Caenorhabditis elegans*, laser ablation has been used to define the functions of many cell types^1^,^2^. However, identification of cells can be challenging and laser ablation experiments are labor intensive, especially for large cells, or complex sets of cells. Genetic cell ablation methods have therefore become increasingly widespread. In *C. elegans*, controlled expression of apoptotic factors has been used to ablate cells^3^,^4^. More recently, the genetically encoded photosensitizers KillerRed (KR) and miniSOG (mini singlet oxygen generator) have been used to ablate cells in *C. elegans*^5^,^6^,^7^. KR and miniSOG both generate reactive oxygen species (ROS) following exposure to high-intensity green or blue light respectively, allowing conditional cell ablation with a high degree of temporal control. Both photosensitizers have also been used for other applications, such as chromophore-assisted light inactivation (CALI)^8^,^9^,^10^ and photodynamic therapy^11^,^12^,^13^,^14^.

KR, derived from the hydrozoan GFP-like protein anm2CP, was the first genetically encoded photosensitizer shown to be effective for cell killing^9^. KR is a type I photosensitizer, generating the ROS superoxide after illumination with green light (585 nm excitation maximum). KR has been used to ablate cells in zebrafish^15^ and more recently in *C. elegans*^6^. Extensive analysis showed that KR could kill a variety of neuron types in *C. elegans*^5^. An advantage of KR is that green light is not toxic to *C. elegans*. However, some limitations of KR have been observed. For example, the superoxide generated by KR can be scavenged by endogenous pathways, possibly accounting for cell type dependent variation in KR killing efficiency^5^. Other researchers have reported toxicity of KR transgenes in the absence of green light irradiation^16^,^17^.

miniSOG, engineered from the LOV domain of *Arabidopsis* phototropin 2, was originally developed for correlative light and electron microscopy^18^. After illumination with blue light (448 nm excitation maximum), miniSOG generates singlet oxygen (^1^O_2_) via a type II reaction^18^, and may also generate other ROS^19^. Expression of cytosolic miniSOG is not generally toxic to cells after blue light illumination. However, miniSOG targeted to the outer mitochondrial membrane results in cell degeneration and death after blue light illumination, presumably due to the generation of toxic levels of ROS at mitochondria^7^. Since its development as a cell killing tool, miniSOG has been used in a variety of ablation experiments^20^,^21^, using blue light exposures of 0.5–1.5 h. The need for extended blue light illumination potentially limits the use of miniSOG as *C. elegans* can be killed by long blue light exposure^22^. Blue light is perceived as noxious by *C. elegans*, in part because blue light generates ROS such as H_2_O_2_^23^. High expression levels of mitochondrially targeted miniSOG may have deleterious effects in the absence of blue light irradiation, due to leaky ROS generation under ambient light^7^.

Here, we demonstrate that membrane targeted miniSOG causes highly efficient ablation of multiple cell types, including neurons, muscles, and the epidermis. The cell killing effects of membrane-targeted miniSOG, like those of mito-miniSOG, are cell autonomous, and result in structural and functional deficits in targeted cells. We also find that a mutant version of miniSOG, previously shown to enhance singlet oxygen generation *in vitro*^24^ can further enhance cell killing efficiency *in vivo*. The combination of these changes yields a greater than ten-fold increase in miniSOG cell killing efficiency compared to mito-miniSOG, without overt deleterious effects in unilluminated animals. We use this enhanced miniSOG to reveal a role for the epidermis in locomotor behavior.

## Results

### Expression of mitochondrial or membrane targeted miniSOG in the epidermis causes paralysis and death

**Figure 1:**
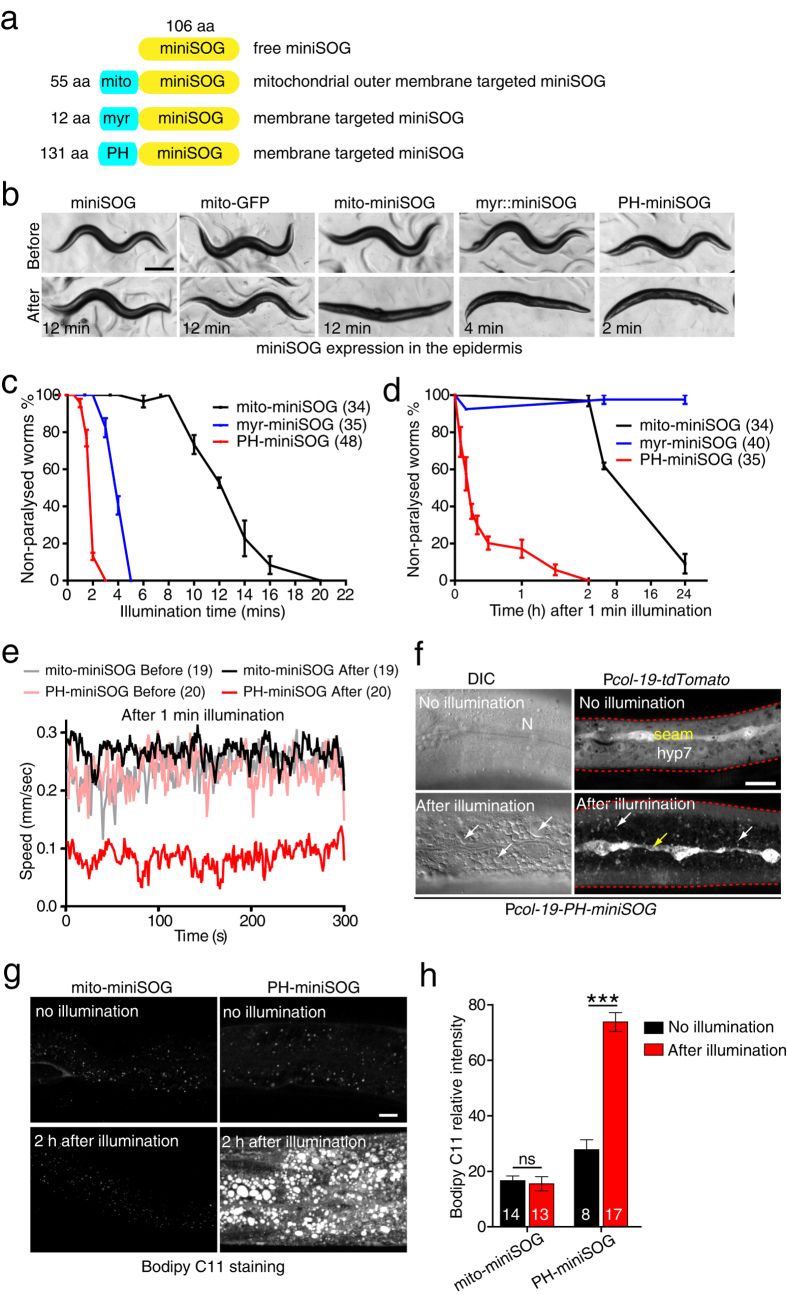
Activation of miniSOG in the epidermis causes paralysis and disrupts epidermal integrity. (**a**) Cartoon of constructs targeting miniSOG to the outer membrane of mitochondria, and to cell membranes. (**b**) Representative images of adult animals immediately before and after blue light illumination. Blue light treatment results in paralysis of mito-miniSOG, myr-miniSOG, and PH-miniSOG expressing animals; times indicate the minimum time for immediate paralysis using 2 Hz blue light illumination. Scale, 250 μm. (**c**) Quantitation of paralysis immediately after blue light illumination, for the indicated times. Numbers are the animals that were analyzed in three independent experiments. (**d**). Quantitation of paralysis at different time points after 1 min blue light illumination. Paralyzed and non-paralyzed animals were counted at specific times after illumination. 4 independent experiments. Numbers are the animals that were analyzed. (**e**) Quantitation of locomotion velocity before and immediately after 1 min blue light illumination at 2 Hz. Transgenic animals were illuminated on 3 cm unseeded plates first and transferred to unseeded plates immediately afterwards for automated worm tracking. Numbers are the animals that were analyzed. (**f**) Representative DIC and confocal images of epidermis before and 4 h after 2 Hz blue light illumination. Images are from live, paralyzed animals expressing P*col-19*-PH-miniSOG. Left, DIC images, N indicates nuclei; arrows indicate vacuoles. Right, epidermal cells were labeled by P*col-19*-tdTomato. Yellow arrow indicates degenerated seam cell and white arrows indicate hyp7 after blue light illumination. Aggregation and decrease of the tdTomato fluorescence signal is evident. Red dash lines indicate epidermal edges. Scale, 10 μm. (**g**) Blue light illumination of PH-miniSOG in the epidermis increases lipid peroxidation. Representative confocal images of Bodipy C11 staining in lateral epidermis. Scale, 10 μm. (**h**) Quantitation of relative Bodipy C11 fluorescence intensity (AU). ***P < 0.001, Student’s t-test. Numbers are the animals that were analyzed.

Our initial goal was to test the effects of mitochondrial miniSOG in epithelial cells such as the epidermis. We expressed miniSOG, fused to the N-terminal 55 amino acids of the outer mitochondrial membrane protein TOMM-20 (hereafter mito-miniSOG)^7^ (Fig. 1a), in the adult epidermis using the *col-19* promoter. As controls, we used animals expressing cytosolic (non-targeted) miniSOG and mito-GFP (Tomm20 targeting). All such transgenic animals were indistinguishable from the wild type in behavior and morphology before illumination (Fig. 1b, Movie S1). After 12 min blue light illumination using an LED source with irradiance ~2 mW/mm^2^, cytosolic miniSOG or mito-GFP epidermal transgenic animals did not display altered behavior or morphology (Fig. 1b, Movie S1). In contrast, mito-miniSOG transgenic animals became paralyzed immediately after blue light illumination (Fig. 1b, Figure S1a, and Movie S2). These animals assumed a linear posture and could not move forwards or backwards, although local muscle twitches could be observed. Such animals eventually died within the next 4–6 hours.

To assess the overall health of epidermal mito-miniSOG transgenic animals, we measured their lifespan when cultured in the dark. We found that P*col-19*-mito-miniSOG expressing animals had a reduced lifespan whereas P*col-19*-miniSOG animals had a normal lifespan (Figure S1b), suggesting overexpression of epidermal mito-miniSOG may have subtly deleterious effects in the absence of illumination. In wild type animals, epidermal mitochondria form a complex tubular network^25^. Epidermal mitochondria appeared abnormal in morphology in mito-miniSOG expressing animals but were normal in animals expressing cytosolic miniSOG (Figure S1c). These observations suggested that mito-miniSOG overexpression can impair epidermal structure and function after blue light illumination, but also had slightly toxic effects in the epidermis in the absence of blue light.

We therefore explored whether other targeting signals could enhance miniSOG toxicity after blue light without affecting organismal health in the absence of irradiation. As membrane targeting had been used in studies of KR mediated cell ablation^5^,^9^, we tested a membrane targeting signal widely used in *C. elegans*, the PH (Pleckstrin Homology) domain from rat PLC-δ^26^ (Fig. 1a). PH domains bind phosphoinositides with high affinity, targeting proteins to the plasma membrane and may also target other organelle membranes (Figure S1d)^27^. As an alternative membrane targeting signal we tested the predicted myristoylation signal sequence from *C. elegans* NCS-2 (Fig. 1a).

After 1 min continuous blue light illumination, 80% of P*col-19*-PH-miniSOG transgenic animals were paralyzed whereas 0% P*col-19*-mito-miniSOG transgenic animals were paralyzed (Figure S1a). As pulsed blue light is more effective than continuous blue light in mito-miniSOG based cell ablation^7^, we tested different frequencies and found 2 Hz (0.25 sec on and 0.25 sec off) to be most effective in paralyzing epidermal PH-miniSOG transgenic animals (Figure S1e). Unless stated, in subsequent experiments we use 2 Hz illumination.

After 12 min blue light exposure 50% of mito-miniSOG transgenic animals were paralyzed, whereas only 2 min blue light was required to paralyze 50% of animals expressing PH-miniSOG at comparable levels (Fig. 1b,c, Movie S2, S3). This result suggested that PH-miniSOG might be more efficient than mito-miniSOG in disrupting epidermal function. Under blue light, 4 min illumination paralyzed 50% of P*col-19*-myr-miniSOG transgenic animals (Fig. 1b,c, Movie S4). 1 min illumination did not immediately paralyze either mito-miniSOG or PH-miniSOG transgenic animals (Fig. 1d), but significantly reduced the locomotion speed of PH-miniSOG transgenic animals (Fig. 1e, Figure S1f). 2 h after illumination, 100% of PH-miniSOG animals were paralyzed (Fig. 1d) and eventually died (defined as nonresponsive to touch) within the next 4–6 h (data not shown). In contrast, a small fraction of myr-miniSOG animals were paralyzed within minutes of 1 min illumination but fully recovered by 24 h later (Fig. 1d), whereas mito-miniSOG animals were superficially wild type immediately after illumination but paralyzed 8 h later and died by 24 h (Fig. 1d). These results suggest mito-miniSOG might have delayed or chronic effects upon blue light illumination, whereas PH-miniSOG has stronger acute effects. PH-miniSOG expressing animals were superficially wild type with normal lifespan, resembling cytosolic miniSOG expressing animals (Figure S1b, Movie S3). The fluorescence levels of membrane-targeted miniSOG were similar to or less than that of mito-miniSOG (Figure S1g,S1h), suggesting the improved killing efficiency of PH-miniSOG or myr-miniSOG does not reflect overall expression level.

The motor deficits and organismal death observed after expression of PH-miniSOG in the epidermis could reflect disruption of epidermal integrity. We examined epidermal morphology using Nomarski differential interference contrast (DIC) microscopy. The hyp7 and lateral epidermal seam cells were superficially wild type in morphology in PH-miniSOG expressing animals without blue light illumination (Fig. 1f). 4 h after 2 min blue light exposure, we observed dramatic changes in epidermal cell morphology, including cytosolic vacuolation and disruption of nuclear shape and position (Fig. 1f, Figure S2a). Cytosolic tdTomato fluorescence became dimmer and aggregated, and seam cells were thinner and degenerated (Fig. 1f). Illumination of animals expressing cytosolic miniSOG in the epidermis did not cause obvious defects in epidermal morphology (not shown). These results suggest PH-miniSOG generated ROS disrupts epidermal structure, leading to degeneration of the epidermis, and eventually organismal death.

As *C. elegans* epidermal cells do not normally undergo apoptosis^28^, we examined further the effects of PH-miniSOG. After blue light illumination, PH-miniSOG expressing animals displayed disrupted epidermal microtubule architecture (Figure S2a). The normally tubular epidermal mitochondria became highly fragmented after illumination (Figure S2b). Damage to the epidermis, as caused by needle or laser wounding, can trigger expression of antimicrobial peptides (AMPs, such as *nlp-29*)^29^,^30^ (Figure S2c). PH-miniSOG expressing animals displayed reduced *nlp-29-GFP* signal 4 h after blue light illumination (Figure S2c), suggesting PH-miniSOG does not simply damage the epidermis but causes epidermal cell death.

Cell death could result from membrane damage due to excessive lipid peroxidation by high levels of ROS at the membrane, as in ferroptosis^31^. To test this hypothesis we examined lipid peroxidation levels *in vivo* using the fluorescent dye C11-Bodipy^32^. We found that lipid peroxidation dramatically increased after blue light illumination of PH-miniSOG animals, but was unchanged from background levels in mito-miniSOG expressing animals (Fig. 1g,h). Together, these data suggest membrane-targeted miniSOG causes a widespread disruption of epidermal cell structure after blue light illumination, potentially due to increased lipid peroxidation leading to membrane damage. Further, the paralysis observed after epidermal disruption reveals a role for the epidermis in locomotion.

### Membrane targeted miniSOG allows highly efficient neuronal ablation

**Figure 2:**
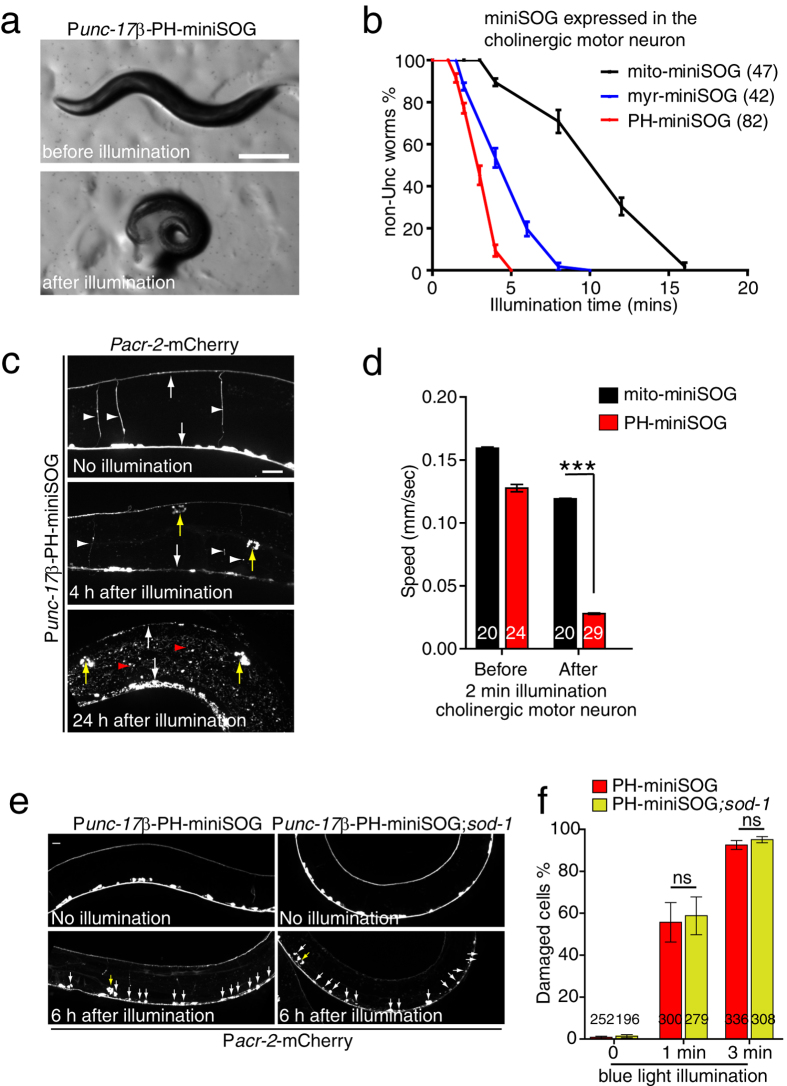
Membrane targeting enhances the efficiency of miniSOG mediated cholinergic neuron ablation. (**a**) Photoablation of cholinergic motor neurons by PH-miniSOG results in uncoordinated movement immediately after 4 min blue light illumination. Scale, 250 μm. (**b**) Quantitation of Unc animals immediately after blue light illumination, for the indicated times. Membrane targeted miniSOG transgenic animals required shorter illumination times to display comparable Unc phenotypes. Numbers are the animals that were analyzed in three independent experiments. (**c**) Morphology of cholinergic motor neurons 4 h and 24 h after 4 min blue light illumination of PH-miniSOG. Motor commissures have disappeared and the remaining dorsal and ventral cord processes have degenerated into puncta. The *unc-17β* and *acr-2* promoters are both expressed in the DA, DB, VA and VB neurons. White arrows indicate ventral and dorsal nerve cords. White arrowheads indicate motor neurons, red arrowheads indicate accumulation of mCherry in the epidermis, and yellow arrow indicates accumulation of mCherry in coelomocytes. Scale, 20 μm. (**d**) Quantitation of locomotion velocity immediately after blue light illumination (2 min, 2 Hz). PH-miniSOG transgenic animal displayed significantly reduced speed compared to mito-miniSOG. Worm locomotion was analyzed by multi worm tracker; in this and subsequent panels n in bars denotes numbers of animals analyzed in three independent experiments. mean ± SEM. ***P < 0.001, t-test. (**e**) Representative morphology of cholinergic motor neurons before and 6 h after 3 min continuous blue light illumination in WT and *sod-1(tm776)* mutant animals expressing P*unc-17β-*PH-miniSOG. Blue light illumination was performed on L3 larvae, and animals imaged 6 h later (early L4 stage). White arrows indicate damaged cell bodies in the ventral nerve cord. The damaged cells eventually died. Yellow arrow indicates accumulation of mCherry in coelomocytes. Scale, 10 μm. (**f**) Quantitation of damaged VNC cholinergic motor neurons 6 h after 1 min and 3 min continuous blue light illumination (see representative images in Panel c) in WT and *sod-1(tm776)* mutant animals. n, number of cells. ns, not significant, Student’s t-test.

To test whether membrane-targeted miniSOG is also more efficient than mito-miniSOG in other cell types, we expressed myr-miniSOG or PH-miniSOG in cholinergic motor neurons using the *unc-17β* promoter. Immediately after 10 min blue light illumination, P*unc-17β* mito-miniSOG adult animals were severely uncoordinated (Unc) and coiled (Fig. 2b), consistent with earlier findings^7^. Both myr and PH membrane targeted miniSOG expressing animals displayed similar Unc phenotypes after blue light illumination, but using significantly shorter exposure times compared to mito-miniSOG (Fig. 2a,b, Movie S5). After 2 min blue light illumination, PH-miniSOG animals displayed significantly reduced locomotion velocity compared to mito-miniSOG animals (Fig. 2d). Normal locomotion was not restored in these animals after 96 h (data not shown), suggesting they had permanently lost neuronal function.

The irreversible behavioral deficits of P*unc-17β* -PH-miniSOG-expressing animals are consistent with cholinergic motor neuron degeneration and death, as previously characterized for mito-miniSOG^7^. To assess how PH-miniSOG affected neuronal morphology we used the P*acr-2*-mCherry marker, expressed in an overlapping set of cholinergic motor neurons^33^. Most neurons displayed obvious degeneration, defined as thinning, vesiculation, or loss of axons, and rounding-up of the cell soma (Fig. 2c, Figure S3a,c). Within 2–4 h of neuronal killing by PH-miniSOG we also observed abundant mCherry aggregates in the epidermis and in coelomocytes (Fig. 2c, Figure S3b,c), suggesting fragments of ablated cells are endocytosed by epidermis and coelomocytes. To assess the efficiency of P*unc-17β*-PH-miniSOG killing at the level of individual neurons we counted damaged cells and found over 90% of cell bodies were damaged 6 h after 3 min blue light illumination (Fig. 2e,f).

In summary, these data suggest PH-miniSOG is significantly more efficient than mito-miniSOG in neuronal ablations. MiniSOG has recently been found to generate ROS other than singlet oxygen *in vitro*^19^. This raises the possibility that cell killing efficiency could be limited by the activity of endogenous detoxification pathways such as superoxide dismutase, as was shown for KR^5^. We found that the efficiency of neuronal and epidermal killing by PH-miniSOG was not enhanced in animals lacking SOD-1, one of the two cytosolic superoxide dismutases^34^ (Fig. 2e,f; Figure S4a,b). These observations suggest that superoxide detoxification may not be limiting for miniSOG killing, consistent with a role for singlet oxygen as the active species.

### Membrane targeting of miniSOG improves killing efficiency in a variety of neurons

**Figure 3:**
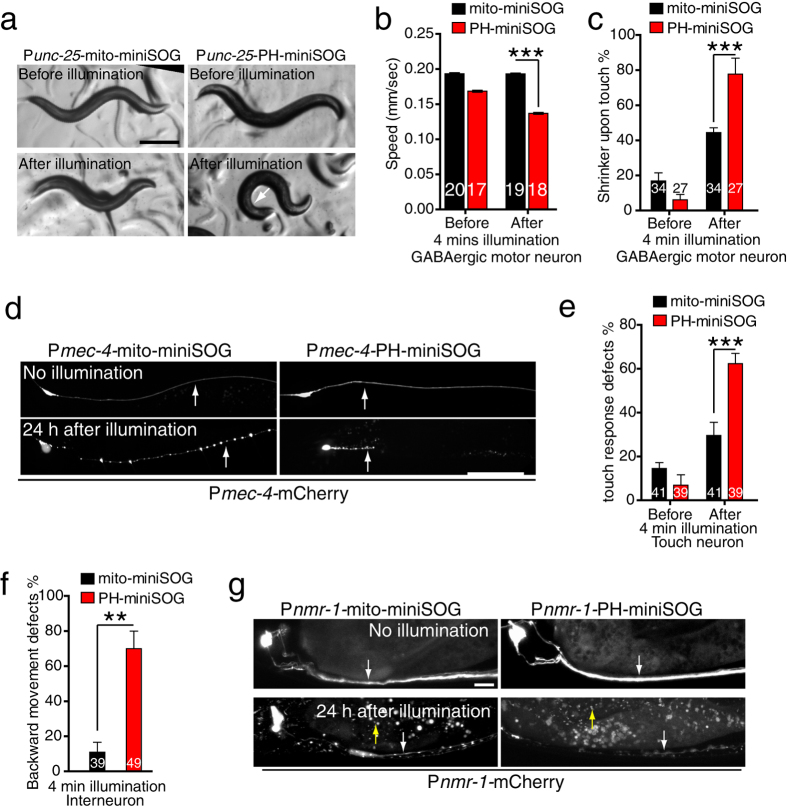
Membrane targeting enhances the efficiency of miniSOG mediated GABAergic, touch, and interneuron ablation. (**a**) P*unc-25*-PH-miniSOG transgenic animals display Unc phenotypes immediately after 4 min blue light illumination, whereas P*unc-25*-mito-miniSOG animals did not. Scale, 250 μm. (**b**) Quantitation of locomotion speed immediately after blue light illumination. P*unc-25*-PH-miniSOG transgenic animals displayed significantly reduced speed compared to P*unc-25*-mito-miniSOG. Numbers are the animals analyzed in 2 independent experiments. ***P < 0.001, t-test. (**c**) Quantitation of shrinker phenotype in P*unc-25*-mito-miniSOG and P*unc-25*-PH-miniSOG expressing animals, before and after blue light illumination (4 min at 2 Hz). n > 30 animals for each transgenic animal. ***P < 0.001, t-test. (**d**) Representative confocal images of PLM neurons before and 24 h after 4 min blue light illumination. The PLM neuron is degenerated in mito-miniSOG and in PH-miniSOG transgenic animals after illumination. Arrows indicate PLM processes. Scale, 20 μm. (**e**) Quantitation of touch responses in P*mec-4*-mito-miniSOG and P*mec-4*-PH-miniSOG before and after blue light illumination. ***P < 0.001, t-test. (**f**) Quantitation of backward movement after head touch in P*nmr-1-*mito-miniSOG and P*nmr-1*-PH-miniSOG transgenic animals after blue light illumination. ***P < 0.001, t-test. (**g**) Representative confocal images of PVC neurons before and 24 h after 4 min blue light illumination. PVC neuron is degenerated in mito-miniSOG and PH-miniSOG transgenic animals after illumination. White arrow indicates PVC neurons and yellow arrows indicate mCherry debris in the epidermis. Scale, 10 μm.

We next tested whether membrane targeting could improve miniSOG’s killing efficiency in other neuron types. We first examined GABAergic motor neurons. Animals expressing PH-miniSOG under the control of the *unc-25*/GAD promoter immediately displayed a shrinker Unc phenotype after 4 min blue light illumination (Fig. 3a, Movie S6) and displayed significantly reduced locomotion speed compared to animals expressing P*unc-25*-mito-miniSOG (Fig. 3b). P*unc-25*-PH-miniSOG animals displayed a more penetrant shrinker phenotype upon nose touch (Fig. 3c), consistent with the higher efficiency of PH-miniSOG over mito-miniSOG.

We then examined mechanosensory neurons, for which mito-miniSOG had not previously been tested. Animals expressing mito-miniSOG or PH-miniSOG in the six touch neurons (*mec-4* promoter) displayed normal touch neuron morphology and mechanosensory behavior prior to blue light illumination (Fig. 3d,e). After 4 min blue light illumination P*mec-4*-PH-miniSOG expressing animals displayed more penetrant touch response defects compared to animals expressing P*mec-4*-mito-miniSOG (Fig. 3e). 24 h later the axons of all touch neurons (ALM, PLM, AVM, and PVM) became punctate and the somas were swollen (Fig. 3d, Figure S5b), consistent with axonal degeneration and cell death.

Finally, we tested the ability of PH-miniSOG to kill interneurons, loss of function in which results in backward movement defects. Expression of PH-miniSOG or mito-miniSOG expression under the control of the *nmr-1* promoter caused interneuron degeneration by 24 h post illumination. P*nmr-1*-PH-miniSOG expressing animals displayed significantly more penetrant backward movement defects immediately after illumination compared to animals expressing P*nmr-1*-mito-miniSOG (Fig. 3f,g). Overall, our data show that PH-miniSOG is more efficient than mito-miniSOG for killing a variety of neuron types.

### Membrane targeted miniSOG allows efficient ablation of muscle cells

**Figure 4:**
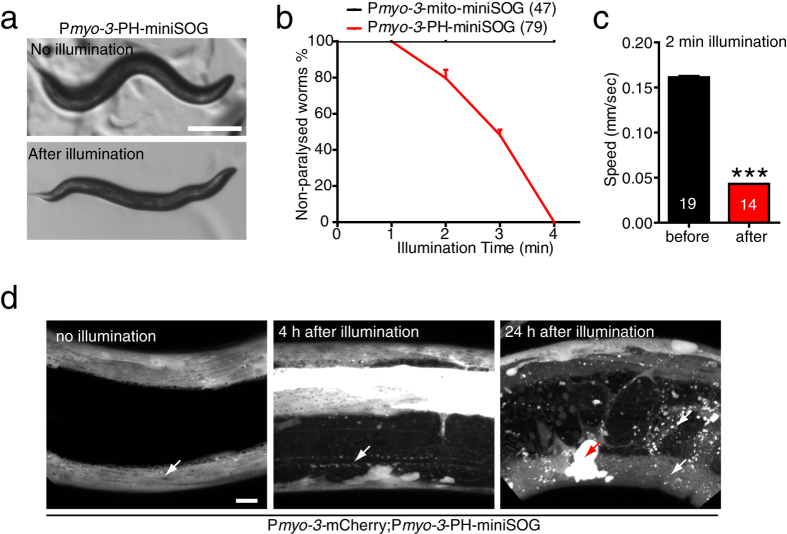
Membrane targeting enhances the efficiency of miniSOG mediated muscle cell ablation. (**a**) Photoablation of body wall muscle by P*myo-3-*PH-miniSOG results in paralysis immediately after blue light illumination. Scale, 250 μm. (**b**) Quantitation of paralysis after blue light illumination. After 4 min illumination, animals that expressed PH-miniSOG in muscle cells were completely paralyzed, while mito-miniSOG expressing animals were not. Numbers are the animals that were analyzed in five independent experiments. (**c**) Quantitation of locomotion velocity immediately after blue light illumination (2 min 2 Hz). P*myo-3*-PH-miniSOG transgenic animals displayed significantly reduced speed compared to P*myo-3-*mito-miniSOG. Multi worm tracker; numbers in bars, n from three independent experiments. Mean + /− SEM. ***P < 0.001, t-test. (**d**) Representative confocal images of muscle cells with and without blue light illumination. Muscle cells degenerated by 4 h after 4 min illumination and did not recover 24 h later in PH-miniSOG transgenic animals. White arrows indicate muscle cell and red arrow indicates mCherry debris in the coelomocytes. Scale, 10 μm.

We then tested whether membrane-targeted miniSOG is also more efficient than mito-miniSOG in muscle cells. Animals expressing PH-miniSOG driven by the *myo-3* promoter were superficially wild type, and after 3 min blue light illumination were completely paralyzed; such paralyzed animals were distinguishable from epidermal miniSOG animals in that they displayed a kinked posture but did not undergo local muscle twitching (Fig. 4a,b, Movie S7). P*myo-3*-mito-miniSOG animals did not show any locomotor defects under similar illumination conditions (Fig. 4b). The locomotion of P*myo-3*-PH-miniSOG expressing animals was also significantly reduced immediately after 2 min illumination (Fig. 4c). Muscle cells in these animals appeared severely damaged as early as 4 h after illumination (Fig. 4d); muscle structure or function were not recovered by 24 h (Fig. 4d). These data show membrane targeted miniSOG is able to kill muscle cells more efficiently than mito-miniSOG.

### Ablation by membrane-targeted miniSOG is cell autonomous

**Figure 5:**
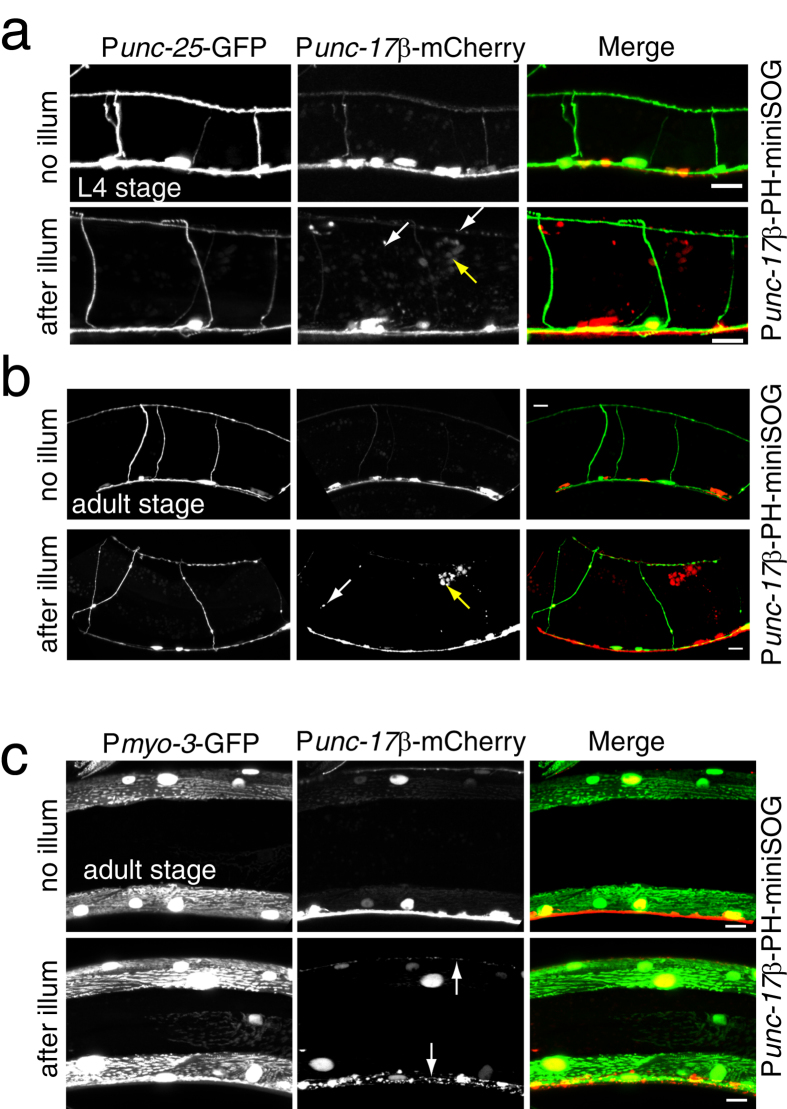
Membrane targeted miniSOG-mediated cell killing is cell autonomous. (**a,b**) GABAergic motor neurons (P*unc-25-*GFP) are intact while cholinergic motor neurons (P*acr-2-*mCherry) degenerate 4 h after blue light illumination in *Punc-17β-*PH-miniSOG transgenic animals. L4 stage (**a**) and adult (**b**) animals were analyzed. White arrows indicate degenerated cholinergic neuron fragments. Yellow arrow indicates mCherry debris in the coelomocytes. Scale, 10 μm. (**c**) Muscle cells (marked by P*myo-3-*GFP*(ccIs4251)*) are intact while cholinergic motor neurons (P*acr-2-*mCherry) degenerate 4 h after blue light illumination in P*unc-17β-*PH-miniSOG transgenic animals. Adult animals were analyzed. White arrows indicate cholinergic neuron fragments. Scale, 10 μm.

Cell ablation by mito-miniSOG has been shown to be cell-autonomous^7^. To test whether membrane-targeted miniSOG ablation is also cell autonomous we examined neurons and muscles located close to PH-miniSOG expressing cells. First, we examined GABAergic motor neurons in animals expressing PH-miniSOG in cholinergic motor neurons. Cholinergic neurons were largely disrupted after blue light illumination (Fig. 5a,b), but nearby GABAergic neurons remained intact (Fig. 5a,b). Activation of miniSOG in the cholinergic motor neurons immediately caused Unc phenotypes, which might also result from damage to muscle cells. We found muscle cells were overall morphologically normal in animals expressing PH-miniSOG in the cholinergic motor neurons (Fig. 5c). PH-miniSOG activation in GABA motor neuron caused their degeneration, but did not appear to alter morphology of adjacent neurons (Figure S5a). Together, these results suggest that membrane targeted miniSOG cell killing is cell-autonomous and does not spread to adjacent tissues.

### The Q103L variant increases miniSOG’s cell killing efficiency

**Figure 6:**
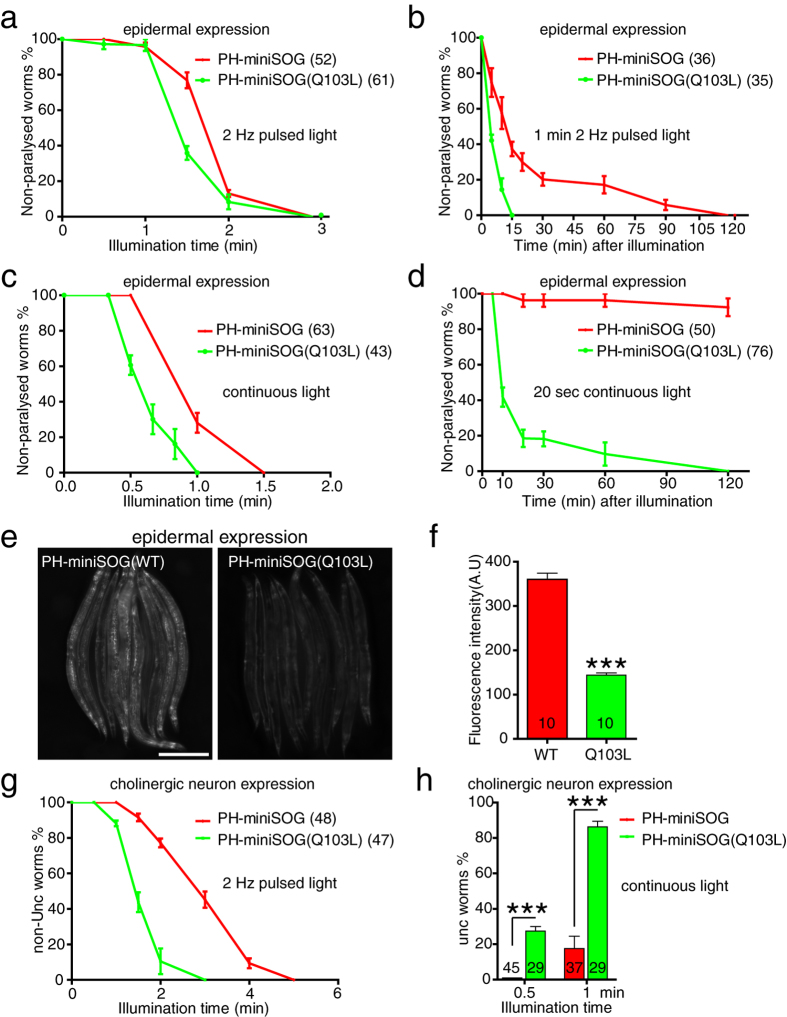
The Q103L variant miniSOG enhances cell ablation efficacy. (**a**) Quantitation of paralysis in animals expressing epidermal PH-miniSOG and PH-miniSOG (Q103L), immediately after blue light illumination (2 Hz) for the indicated times. (**b**) Onset of paralysis in animals expressing epidermal PH-miniSOG or PH-miniSOG (Q103L), after 1 min 2 Hz blue light illumination. (**c**) Quantitation of paralyzed PH-miniSOG and PH-miniSOG(Q103L) animals immediately after continuous blue light illumination, for the indicated times. (**d**) Onset of paralysis in animals expressing epidermal PH-miniSOG and PH-miniSOG (Q103L) animals after 20 sec continuous blue light illumination. (**e**) Green fluorescence images of P*col-19*-PH-miniSOG(WT) and P*col-19*-PH-miniSOG(Q103L) transgenic animals, identical exposures. Scale, 250 μm. (**f**) Quantitation of green fluorescence intensity in panel e. ***P < 0.001, t-test. Numbers are the animals that were analyzed. (**g**) Quantitation of Unc animals immediately after 2 Hz blue light illumination, for the indicated times. PH-miniSOG and PH-miniSOG(Q103L) were expressed under the control of *unc-17β* promoter. (**h**) Quantitation of Unc animals after 0.5 and 1 min continuous blue light illumination.

Recently, a mutant version of miniSOG in which a glutamine is replaced by a leucine has been found to result in a higher quantum efficiency of singlet oxygen production *in vitro*^24^. We tested this mutant (Q103L in our residue numbering) in our cell ablation assays. Animals expressing PH-miniSOG(Q103L) in the epidermis were superficially wild type in the absence of blue light illumination (Figure S6a). After 2 min illumination, the WT and Q103L animals displayed similar rates (~90%) of acute paralysis (Fig. 6a). However, after 1.5 min blue light, the Q103L variant caused a slight increase in acute paralysis; ~70% of PH-miniSOG(Q103L) animals were paralyzed, compared to 20% of PH-miniSOG (WT) animals (Fig. 6a), suggesting that the Q103L variant may be slightly more effective than WT miniSOG. We then examined the longer-term effects of brief blue light exposures. Notably, after 1 min blue light illumination, the PH-miniSOG(Q103L) animals became completely paralyzed within 15 min, whereas PH-miniSOG(WT) were not fully paralyzed until 2 h post illumination (Fig. 6b). PH-miniSOG(Q103L)-expressing animals were also slightly more sensitive to continuous light (Fig. 6c). We exposed both types of transgenic animals to 20 sec continuous blue light, which did not result in acute paralysis. However PH-miniSOG(Q103L) animals became completely paralyzed within 2 h of illumination and did not recover (Fig. 6d), whereas PH-miniSOG animals were slightly slow-moving (Fig. 6d), and were superficially wild type 24 h later (data not shown). Animals expressing the Q103L variant were also more sensitive to illumination with 30 sec pulsed light (Figure S6b). These results suggest the Q103L mutation enhances the efficacy of miniSOG in epidermal cell killing.

A possible explanation for the stronger cell killing of the Q103L variant is that its expression or stability could be higher than miniSOG(WT). Yet, miniSOG(Q103L) transgenic animals showed significantly lower fluorescence intensity than miniSOG(WT) (Fig. 6e,f). The increase in efficacy of the Q103L variant is thus unlikely to be due to differences in expression, but rather by the increased production of singlet oxygen^24^.

We then tested whether miniSOG(Q103L) enhanced cell killing ability in cholinergic motor neurons. P*unc-17β*-PH-miniSOG(Q103L) transgenic animals displayed normal locomotion (Figure S6c). After 2 min pulsed light approximately 90% of P*unc-17β-*PH-miniSOG(Q103L) animals displayed uncoordinated movement, whereas a comparable phenotype takes 4 min for PH-miniSOG(WT) (Fig. 6g). 30 sec of continuous blue light illumination had no acute effect on P*unc-17β-*miniSOG(WT) animals but caused ~30% of P*unc-17β-*miniSOG(Q103L) animals to be Unc (Fig. 6h). Similarly, 1 min illumination resulted in 90% of P*unc-17β-*miniSOG(Q103L) animals being immediately Unc, compared to less than 20% of P*unc-17β-*miniSOG(WT) transgenic animals (Fig. 6h). 20 sec continuous blue light did not result in acute Unc phenotypes in either transgenic strain, but within 2 h of exposure 60% P*unc-17β-*PH-miniSOG(Q103L) animals became completely Unc, and did not recover normal locomotion (Figure S6d), whereas only 10% P*unc-17β-*PH-miniSOG(WT) animals were Unc within 2 h (Figure S6d). These results suggest the Q103L mutant miniSOG enhances the acute and delayed toxic effects of miniSOG expression.

## Discussion

The original mitochondrially targeted miniSOG allowed effective optogenetic cell ablation in *C. elegans*, but often required long illumination times to achieve full cell killing^7^. Moreover, epidermal mito-miniSOG expression alters mitochondrial morphology (this study), an effect that might be less readily detected in neurons. This effect may in part reflect overload of the mitochondrial import apparatus and in part the effects of low levels of ROS generated in the absence of blue light. The aberrant mitochondrial morphology and decreased lifespan of epidermal mito-miniSOG-expressing animals prompted this investigation into alternative ways to target miniSOG. We find that the combination of membrane targeting and the Q103L point mutation allows highly efficient miniSOG mediated ablation, requiring much shorter illumination times. Importantly, the faster cell killing remains cell autonomous, permanent, and in the absence of blue light illumination the killing transgenes appear to have no toxic effects. As blue light is toxic to *C. elegans*, these improvements should significantly enhance the utility of miniSOG as an optogenetic tool.

Membrane targeting by the PH domain was more efficient in cell disruption than mito-miniSOG for all cell types tested (epidermis, neurons, and muscle). In the epidermis and cholinergic neurons, membrane targeting by myristoylation was also more effective than mito-miniSOG, but not quite as effective as PH-miniSOG. We note that in our analysis of cholinergic, GABAergic, and interneuron killing we used mito-miniSOG transgenes generated at DNA concentrations 2.5-fold higher than the PH-miniSOG transgenes made here. These comparisons may thus slightly underestimate the relative efficiency of PH-miniSOG. Our comparisons of epidermal, muscle, and touch neuron disruption used transgenes that were generated at similar DNA concentrations and suggest that PH-miniSOG is approximately 5–6 times as potent as mito-miniSOG.

The PH domain used here confers plasma membrane targeting in *C. elegans* embryos^26^, but in the differentiated epidermis also localizes to membrane associated puncta (Fig S1d), possibly corresponding to stacks of invaginated apical plasma membrane frequently seen in EM sections of the epidermis. Myristoylation sequences also target primarily to the plasma membrane^35^, but were less effective in our assays, suggesting plasma membrane targeting may not fully account for the enhanced effects of PH-miniSOG. Other membrane targeting signals have been used for KR in zebrafish^15^, and could be tested in future work, although in *C. elegans* cell killing by myristoylated KR had efficacy similar to that of cytosolic KR^5^.

A significant enhancement in miniSOG killing efficiency resulted from the missense mutation Q103L. This mutation was engineered to reduce hydrogen bonding between the miniSOG protein and its flavin mononucleotide (FMN) chromophore, reducing the competing electron transfer reaction to FMN and allowing greater electron transfer to generate singlet oxygen^24^. *In vitro*, the Q103L variant displays about a 5-fold increase in quantum efficiency of singlet oxygen production. Although we have not yet been able to measure singlet oxygen in *C. elegans in vivo*, our observations in the context of membrane targeted miniSOG show that the Q103L change causes a small, but significant, increase in cell killing efficiency, more pronounced when the delayed effects of miniSOG are examined. Additional evolution of miniSOG for improved quantum yield may further enhance cell killing efficacy.

ROS generation by miniSOG triggers a sequence of cellular changes that result in cell degeneration and death. These changes may differ depending on the cell type and the subcellular targeting of miniSOG. Previous analysis indicated that mito-miniSOG induced neuronal death was independent of the apoptotic or phagocytic pathways^7^. The effects of membrane targeted miniSOG resemble those of mito-miniSOG, but might be triggered in different ways. Membrane damage by photosensitizers is thought to cause loss of cell integrity and necrosis, due to the oxidation of lipid bilayers^36^. Membrane miniSOG induced death may be the result of a similar non-physiological cell disruption. Elevated membrane lipid peroxidation has been observed in a non-apoptotic cell death pathway known as ferroptosis^31^. Although ferroptosis has not been reported in *C. elegans*, membrane-targeted miniSOG could cause cell death by a related mechanism. The absence of functional recovery after ablations is consistent with irreversible cell damage, and argues against functional compensation from other cells or from regenerative pathways.

Membrane-targeted miniSOG transgenic animals appear superficially normal under standard culture conditions (ambient light, room temperature), suggesting membrane targeted miniSOG is not strongly toxic in the absence of blue light. As a precaution in this work, we reared animals in the dark, but this does not appear to be necessary for routine experiments. Although the enhanced miniSOG killing minimizes use of blue light, it should be kept in mind that blue light exposure has complex effects on *C. elegans* behavior^22^,^23^,^37^. Analysis of miniSOG-ablated animals should always be precisely controlled for the effects of blue light exposure. The use of PH-miniSOG raises a further concern of whether ROS generated close to the cell surface might have cell-non-autonomous effects. However, our analysis suggests PH-miniSOG mediated cell ablation does not damage adjacent cells. While the cell degeneration and killing effects of PH-miniSOG appear to be cell autonomous, we note that after neuronal or muscle killing, cellular debris accumulated in epidermis and coelomocytes, consistent with the idea that the epidermis is responsible for clearance of cell fragments^28^,^38^. Activation of such non-autonomous clearance pathways is therefore an expected consequence of the widespread cell fragmentation induced by optogenetic cell killing.

We find that optogenetic disruption of the epidermis causes acute paralysis. These findings echo an early observation that ectopic expression of the MEC-4 DegENaC channel in the epidermis caused abnormal movement^39^ and suggest a role for the epidermis in locomotion. The epidermis has numerous physiological functions in the adult^28^. Importantly, the paralysis observed after epidermal disruption was distinguishable from that caused by ablation of body wall muscles (compare Movie S3 and S7), suggesting epidermally disrupted animals are not paralyzed simply due to a secondary disruption of muscle function. The epidermis physically links the musculature and the exoskeleton (cuticle), and epidermal ion transport regulates the function of the motor circuit^40^. As epidermally disrupted animals display local, uncoordinated muscle contractions, one model is that in such animals muscles are able to contract but are unable to transduce force through the epidermis. However, disruption of the epidermis may cause additional functional deficits in the locomotor circuit. The increased efficiency in cell killing allowed by membrane targeted miniSOG should facilitate its use in *C. elegans* and may also be relevant to applications in photodynamic therapy.

## Materials and Methods

### *C. elegans* genetics and transgenes

*C. elegans* strains were maintained on nematode growth medium (NGM) plates seeded with *E. coli* OP50 at 22 °C except when otherwise noted. All miniSOG transgenic animals described here appear superficially wild type in behavior and morphology in the absence of blue light illumination. In general, miniSOG transgenic strains can be propagated in normal ambient light (on the lab bench or incubator). For all experiments (unless otherwise stated) in this paper, we grew dark-reared animals by picking parental animals in the L4 stage and keeping them in foil-covered boxes until the experimental manipulation.

Plasmids and strains used in this study are listed in Table S1. Comparisons of miniSOG variants in the epidermis used transgenic arrays made by injection of similar concentrations of test DNA. Neuronally expressed mito-miniSOG arrays were generated at 50 ng/μl, compared to 20 ng/μl for membrane miniSOG. At least 2 transgenes were tested per experiment; representative arrays are listed in Table S1.

### Blue light illumination

We used a blue LED (UHP-MIC-LED-460) light source (460 ± 5 nm, spectrum half width 27 nm, Prizmatix, Givat Shmuel, Israel) for illumination as described^10^. Briefly, the irradiance at the specimen was 2.01 mW/mm^2^ as measured using a D10MM power sensor connected to a PM50 Optical Power Meter (Thorlabs, Newton, New Jersey, US). Pulse frequency was controlled by a digital function generator/amplifier (PI-9587C, PASCO, Roseville, CA). The light source was ~10 cm above the plate surface. To illuminate animals, we transferred 15–20 young adults to a 3 cm unseeded NGM plate poured with 60 μl of 100 mM CuCl_2_ on the rim of the plate. The blue light illumination covers the entire plate surface. Overall, the irradiance of our LED setup is approximately 3.5× higher than that of the epifluorescence setup tested previously and has a slightly different excitation spectrum^7^. After illumination, animals were kept in the dark until analysis.

### Behavior analysis and quantitation

Animals were defined as paralyzed if they were unable to move forward or backward, whether or not they displayed any local or ineffective muscle contractions. We used a multi-wormtracker to measure locomotion speed^41^. Touch response assays were performed as previously described^42^. Each animal was tested by touching the head five times and by touching the tail five times, and the data combined for statistical analysis. The shrinker Unc phenotype (for P*unc-25*-PH-miniSOG) and backward movement Unc phenotype (P*nmr-1*-PH-miniSOG) were measured after head touch with worm pick. To quantitate miniSOG induced paralysis or other locomotor phenotypes, at least three independent experiments (10–15 animals each) were performed.

### Bodipy C11 staining

Epidermal mito-miniSOG and PH-miniSOG transgenic animals were illuminated with blue light for 2 min then after 1.5 h were transferred into 10 μM Bodipy 581/591 C11 solution and stained for 30 min. Non-illuminated transgenic animals served as controls. Animals were washed with M9 for three times and imaged with Zeiss 710 confocal microscope using a 561 nm excitation laser and LP 590 nm emission filters. Relative fluorescence intensity was quantified using MetaMorph.

### Imaging

For fluorescence imaging of epidermal and neuronal cells, young adult worms were anesthetized with 12 mM levamisole on 2% agar pads. Z-stack images were captured either on a Zeiss LSM710 confocal microscope with 63×, NA 1.4 objective or on an ANDOR spinning disk confocal microscope with 63×, NA 1.4 objective with an ANDOR iXon Ultra camera. Maximum intensity projections and adjustment of brightness and contrast were performed using Metamorph software. Whole animal fluorescence images were taken using a Zeiss Diskovery V12 dissecting stereomicroscope with Nikon DS Qi-1 monochrome camera. Nomarski DIC images were taken on a Zeiss Axioplan 2 microscope using a 100×, NA 1.4 oil objective.

### Statistics

All statistical tests used GraphPad Prism. Two-way comparisons used Student’s t test. Survival proportions used curve comparison. For multiple comparisons, we used one-way ANOVA.

## Additional Information

**How to cite this article**: Xu, S. and Chisholm, A. D. Highly efficient optogenetic cell ablation in *C. elegans* using membrane-targeted miniSOG. *Sci. Rep.* **6**, 21271; doi: 10.1038/srep21271 (2016).

## Supplementary Material

Supplementary Information

Supplementary Movie S1

Supplementary Movie S2

Supplementary Movie S3

Supplementary Movie S4

Supplementary Movie S5

Supplementary Movie S6

Supplementary Movie S7
