# Supplementary material for: Highly efficient optogenetic cell ablation in C. elegans using membrane-targeted miniSOG
Source: Sci Rep. 2016 Feb 10;6:21271. doi: 10.1038/srep21271 (PMC4748272; doi:10.1038/srep21271)
Supplement: Supplementary Information [file srep21271-s1.pdf]

Highly efficient optogenetic cell ablation in *C. elegans* using membrane-targeted miniSOG

Suhong Xu* and Andrew D. Chisholm*

Division of Biological Sciences, Section of Cell and Developmental Biology, University of California, San Diego, 9500 Gilman Drive, La Jolla, CA 92093

*Correspondence to: suhongxu@gmail.com (S.X.) and chisholm@ucsd.edu (A.D.C.)

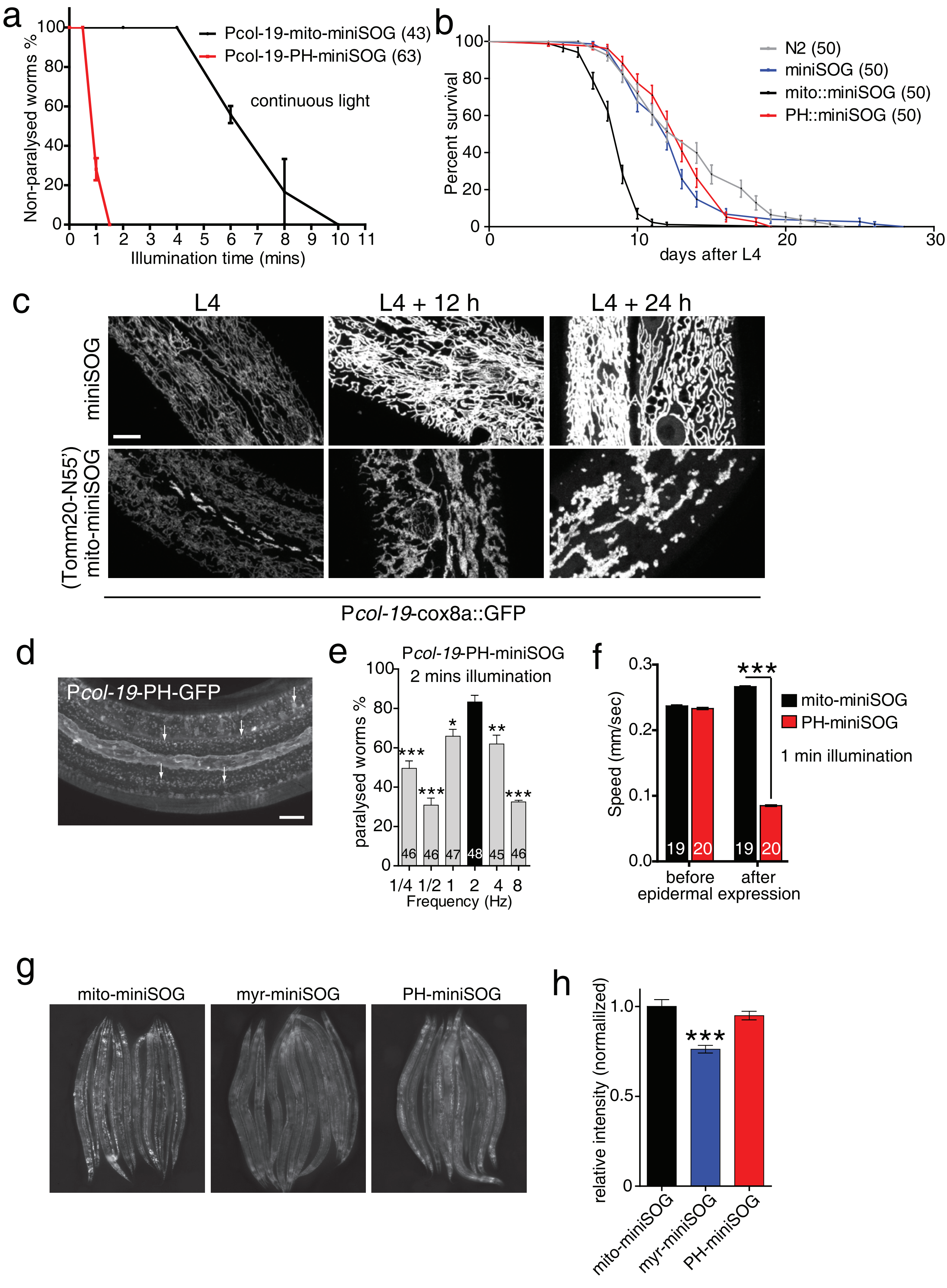
a
Non-paralysed worms %
Illumination time (mins)
Pcol-19-mito-miniSOG (43)
Pcol-19-PH-miniSOG (63)
continuous light
b
Percent survival
days after L4
N2 (50)
miniSOG (50)
mito::miniSOG (50)
PH::miniSOG (50)
c
L4
L4 + 12 h
L4 + 24 h
miniSOG
(Tomm20-N55') mito-miniSOG
Pcol-19-cox8a::GFP
d
Pcol-19-PH-GFP
e
Pcol-19-PH-miniSOG
2 mins illumination
paralysed worms %
Frequency (Hz)
f
Speed (mm/sec)
mito-miniSOG
PH-miniSOG
1 min illumination
before
after
epidermal expression
g
mito-miniSOG
myr-miniSOG
PH-miniSOG
h
relative intensity (normalilzed)
mito-miniSOG
myr-miniSOG
PH-miniSOG

Figure S1

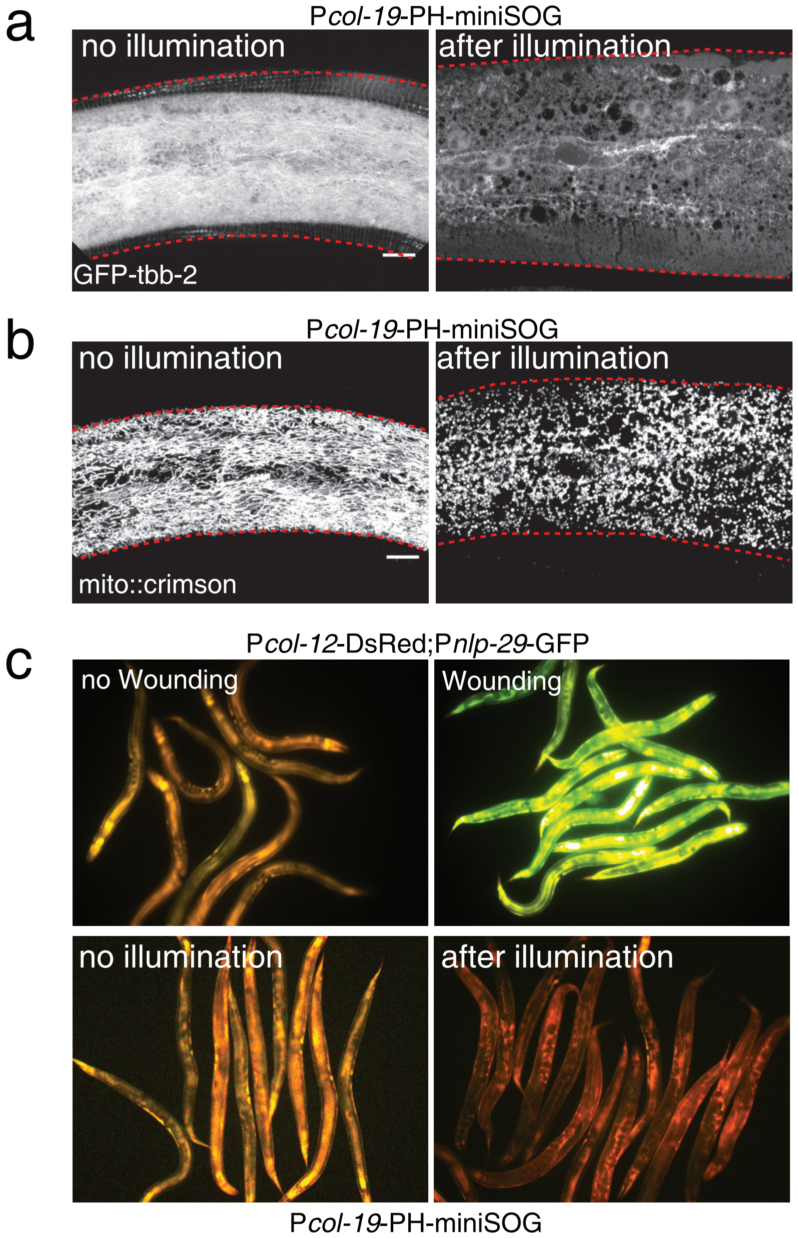
a
Pcol-19-PH-miniSOG
no illumination
after illumination
GFP-tbb-2
b
Pcol-19-PH-miniSOG
no illumination
after illumination
mito::crimson
c
Pcol-12-DsRed;Pnlp-29-GFP
no Wounding
Wounding
no illumination
after illumination
Pcol-19-PH-miniSOG

Figure S2

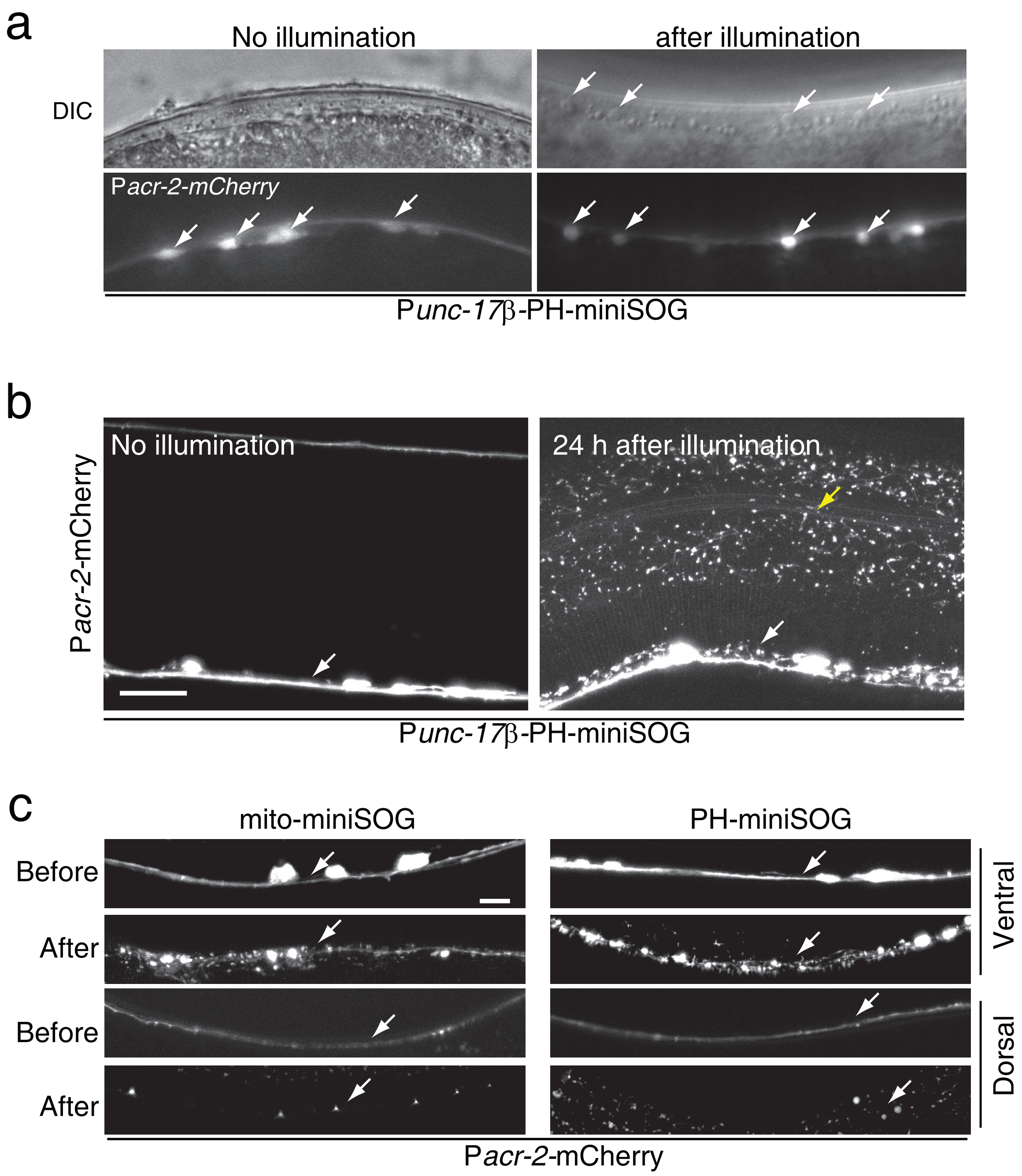
a
No illumination
after illumination
DIC
Pacr-2-mCherry
Punc-17β-PH-miniSOG
b
Pacr-2-mCherry
No illumination
24 h after illumination
Punc-17β-PH-miniSOG
c
mito-miniSOG
PH-miniSOG
Before
After
Before
After
Ventral
Dorsal
Pacr-2-mCherry

Figure S3

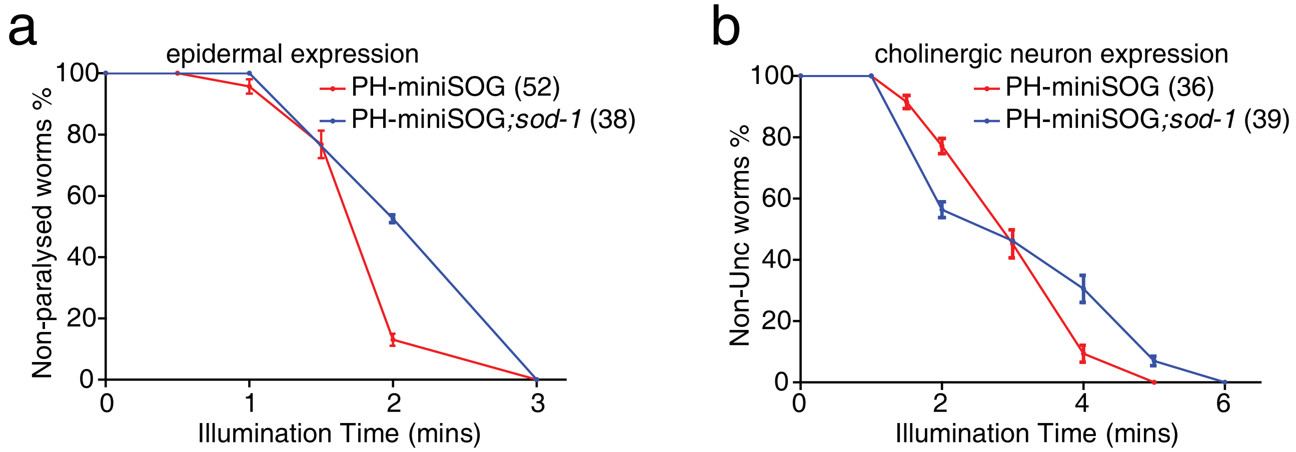
a
epidermal expression
PH-miniSOG (52)
PH-miniSOG;*sod-1* (38)
Non-paralysed worms %
0
20
40
60
80
100
0
1
2
3
Illumination Time (mins)
b
cholinergic neuron expression
PH-miniSOG (36)
PH-miniSOG;*sod-1* (39)
Non-Unc worms %
0
20
40
60
80
100
0
2
4
6
Illumination Time (mins)

Figure S4

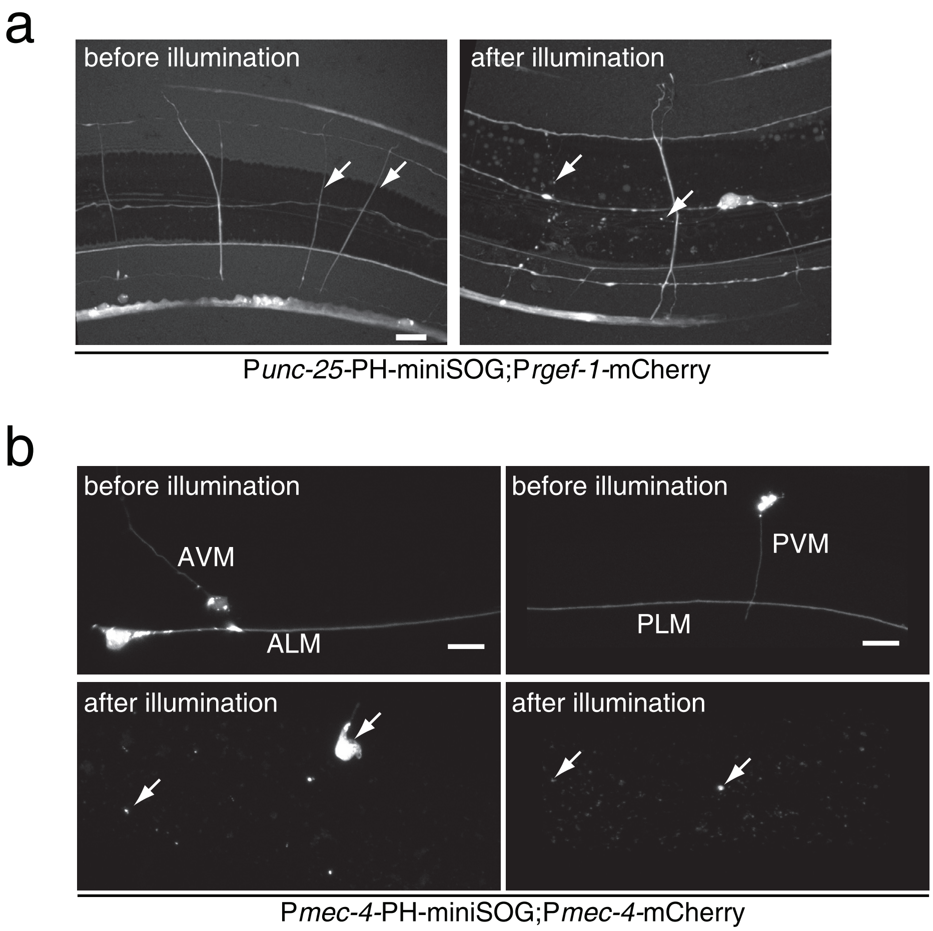
a
before illumination
after illumination
Punc-25-PH-miniSOG;Prgef-1-mCherry
b
before illumination
AVM
ALM
before illumination
PVM
PLM
after illumination
after illumination
Pmec-4-PH-miniSOG;Pmec-4-mCherry

Figure S5

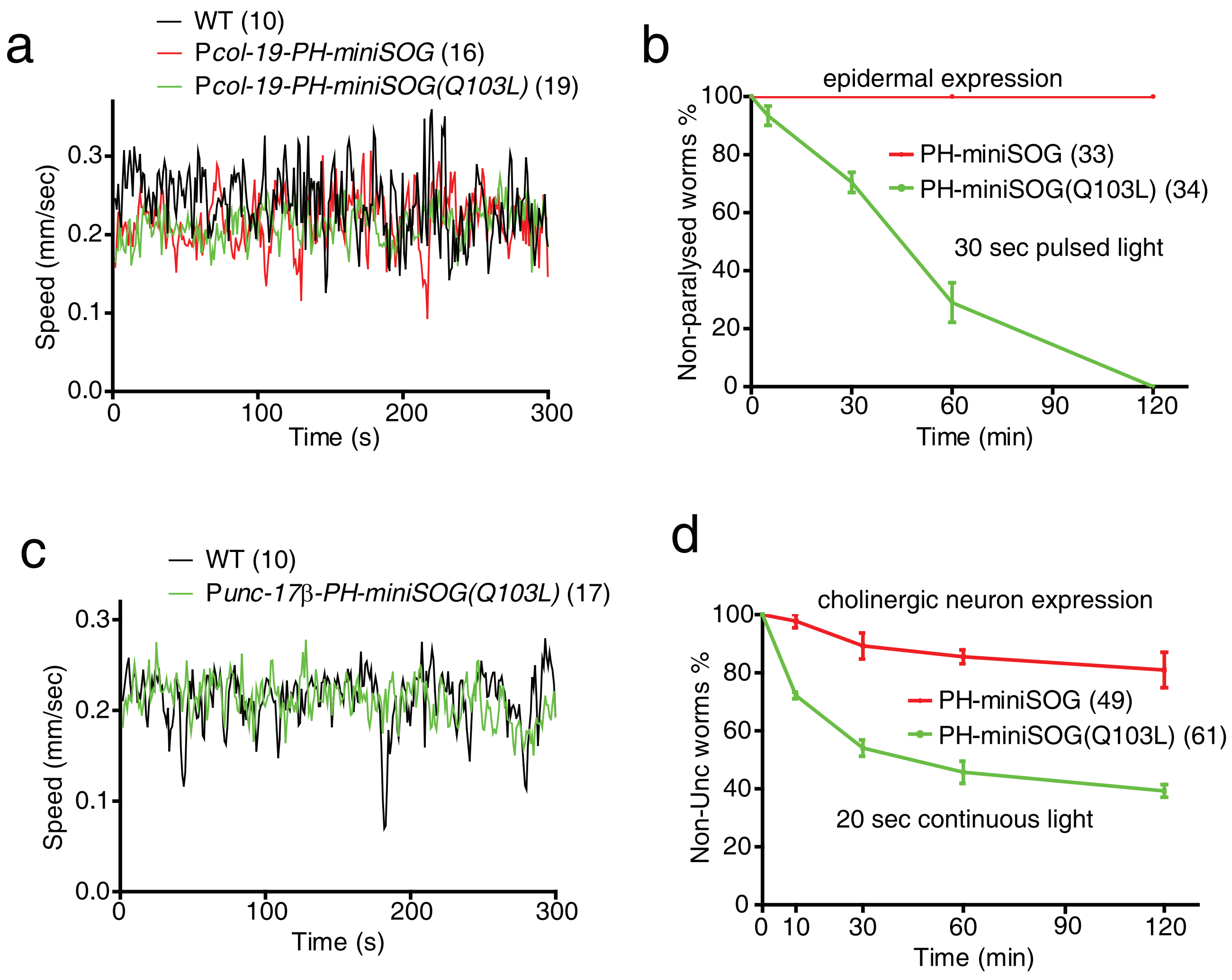
a
WT (10)
Pcol-19-PH-miniSOG (16)
Pcol-19-PH-miniSOG(Q103L) (19)
Speed (mm/sec)
Time (s)
b
epidermal expression
PH-miniSOG (33)
PH-miniSOG(Q103L) (34)
30 sec pulsed light
Non-paralysed worms %
Time (min)
c
WT (10)
Punc-17β-PH-miniSOG(Q103L) (17)
Speed (mm/sec)
Time (s)
d
cholinergic neuron expression
PH-miniSOG (49)
PH-miniSOG(Q103L) (61)
20 sec continuous light
Non-Unc worms %
Time (min)

Figure S6

Supplemental Information

Table S1. New miniSOG transgenic strains and plasmids

Cell types	Strains	Genotype/Transgenes[1]	Plasmid [2]	Concentration of miniSOG constructs
Epidermis	CZ16052	P*col-19-miniSOG (juEx4532)*	pCZGY2137	10 ng/μl
	CZ16855	P*col-19-cox8-miniSOG(juEx4973)*	pCZGY2136	10 ng/μl
	CZ16054	P*col-19-mito-miniSOG (juEx4534)*	pCZGY2138	10 ng/μl
	CZ23271	P*col-19-myr-miniSOG (juEx7095)*	pCZGY2847	10 ng/μl
	CZ16493	P*col-19-PH-miniSOG (juEx4771)*	pCZGY2141	10 ng/μl
	CZ16236	P*col-19-PH-miniSOG/Pcol-19-tdTomato (juIs400)*	PCZGY2141/ pCZGY1575	10 ng/μl of each
	CZ23277	P*col-19-PH-miniSOG(Q103L) (juEx7101)*	pCZGY2850	10 ng/μl
Body wall muscle	CZ15071	P*myo-3-mito-miniSOG/Pmyo-3-mCherry (juEx4076)*	pCZGY1555/ pCZGY872	1 ng/μl of each
	CZ22703	P*myo-3-PH-miniSOG/*P*myo-3-mCherry (juEx6916)*	pCZGY2838/ pCZGY872	5 ng/μl of each
Cholinergic motor neuron	CZ15033/ CZ15844	P*unc-17β-mito-miniSOG*/P*acr-2-mCherry (juIs385)*	pCZGY1558/ pCZGY847	50 ng/μl of each
	CZ23273	P*unc-17β-myr-miniSOG*/P*acr-2-mCherry (juEx7097)*	pCZGY2848/ pCZGY847	20 ng/μl of each
	CZ22692	P*unc-17β-PH-miniSOG/* P*acr-2-mCherry (juEx6905)*	pCZGY2844/ pCZGY847	20 ng/μl of each
	CZ23279	P*unc-17β-PH-miniSOG(Q103L)/* P*acr-2-mCherry (juEx7103)*	pCZGY2851/ pCZGY847	20 ng/μl of each
GABAergic motor neuron	CZ19758	P*unc-25-mito-miniSOG*/P*unc-25-crimson (juEx5983)*	pSK9/pSK10	50 ng/μl of each
	CZ22698	P*unc-25-PH-miniSOG*/P*rgef*-1-*mCherry (juEx6911)*	pCZGY2842/ pCZGY922	20 ng/μl of each

Interneuron	CZ14478	P*nmr-1-mito-miniSOG*/P*nmr-1-mCherry (juEx3771)*	pCZGY1552/ pCZGY903	50 ng/μl of each
	CZ22695	P*nmr-1-PH-miniSOG*/P*nmr-1-mCherry (juEx6908)*	pCZGY2845/ pCZGY903	20 ng/μl of each
Touch neuron	CZ23283	P*mec-4-mito-miniSOG*/P*mec-4-mCherry (juEx7107)*	pCZGY2852/ pCZGY546	20 ng/μl of each
	CZ23281	P*mec-4-PH-miniSOG*/ P*mec-4-mCherry (juEx7105)*	pCZGY2840/ pCZGY546	20 ng/μl of each
Microtubule and mitochondria marker	CZ21790	P*col-19-GFP-tbb-2*;P*col-19-mito-crimson (juEx6578)*	pCZ899/pCZ GY2160	10 ng/μl of each

Notes:

1. Co-injection markers were P*ttx-3-GFP* or P*ttx-3-RFP* (50 ng/μl)

2. All miniSOG clones used DNA codons optimized for mammals [1].

Supplemental Figure Legends

Figure S1. Comparison of the effects of mito-miniSOG and PH-miniSOG in epidermis

a. Quantitation of paralysis in epidermal mito-miniSOG and PH-miniSOG expressing animals immediately after continuous blue light illumination for the indicated period. All transgenes use *col-19* promoter. Numbers are the animals that were analyzed in four independent experiments.

b. Lifespan of wild type and miniSOG transgenic animals. Only mito-miniSOG transgenics displayed significantly reduced lifespan compared to the WT. $p < 0.001$, survival test, error: 95% CI. $n = 50$ animals for each strain.

c. Mito-miniSOG disrupts the tubular structure of mitochondria in the epidermis during development. Representative confocal images of epidermal mitochondria (P*col-19*-mito-GFP) in transgenic animals expressing cytosolic miniSOG and mito-miniSOG at different stages. Scale, 10 μm.

d. The PH domain targets fusion proteins to the epidermal cell membrane. Representative confocal image of PH-GFP expressed in the epidermis. Arrows indicate apical puncta. Scale, 10 μm.

e. Quantitation of paralysis in P*col-19*-PH-miniSOG animals immediately after 2 min blue light illumination at different frequencies. Numbers are the animals that were analyzed in three independent experiments. *, $P < 0.05$, **, $P < 0.01$, ***, $P < 0.001$, One-way ANOVA.

f. Quantitation of locomotion velocity immediately after blue light illumination. P*col-19*-PH-miniSOG transgenic animals displayed significantly reduced speed compared to P*col-19*-mito-miniSOG transgenics. Numbers are the animals that were analyzed in 2 independent experiments. ***, $P<0.001$, t-test.

g. Green fluorescence images of epidermal miniSOG transgenic animals. Scale, 250 μm.

h. Quantitation of green fluorescence intensity in panel f. ***, $P < 0.001$, One way ANOVA. mito-miniSOG fluorescence intensity was normalized to 1.

Figure S2. Membrane targeted miniSOG expressed in the epidermis causes cell disruption after blue light illumination.

a. Animals expressing P*col-19*-PH-miniSOG the epidermis display disrupted microtubule structure (P*col-19*-GFP-TBB-2 marker) after 2 min blue light illumination. Red dashed lines indicate the outlines of the animal. Scale, 10 μm.

b. Animals expressing PH-miniSOG in the epidermis display disrupted mitochondrial structure (P*col-19*-mito::crimson) after 2 min blue light illumination. Scale, 10 μm.

c. PH-miniSOG activation in the epidermis does not induce antimicrobial peptide expression (P*nlp-29*-GFP reporter *frIs7*, containing P*col-12*-dsRed internal control). Top: needle wounding triggers P*nlp-29*-GFP induction in the epidermis 4 h later. Bottom: PH-miniSOG expression in the epidermis paralyzed animals but did not induce P*nlp-29*-GFP*,* 4 h after 2 min blue light illumination.

Figure S3 Effects of PH-miniSOG on cholinergic neuron morphology

a. DIC and fluorescence images of P*unc-17β*-PH-miniSOG transgenic animals before and 2 h after illumination (2 min and 2 Hz). Arrows indicate rounded-up soma in the VNC in the DIC images, and P*acr-2-mCherry* in the fluorescence image.

b. Representative confocal images of *Pacr-2-mCherry*; P*unc-17*β-PH-miniSOG before and 24 h after blue light illumination. White arrows indicate VNC and yellow arrowheads indicate mCherry aggregates in the epidermis. Scale, 10 μm.

c. Representative confocal images of *Pacr-2*-mCherry before and 24 h after blue light illumination. Both mito-miniSOG and PH-miniSOG (under the control of *unc-17β* promoter) induce cholinergic neuron degeneration after blue light illumination. White arrows indicate VNC and DNC. Scale, 10 μm.

Figure S4. Membrane targeted miniSOG-mediated cell killing is not enhanced in superoxide dismutase *sod-1* mutant

a. Quantitation of paralysis in WT and *sod-1(tm776)* mutant animals expressing epidermal PH-miniSOG immediately after 2 Hz blue light illumination, for the indicated times. Note *sod-1* mutant does not enhance the paralysis of PH-miniSOG transgenic animals after blue light illumination.

b. Quantitation of Unc animals in WT and *sod-1(tm776)* mutant expressing PH-miniSOG immediately after 2 Hz blue light illumination for the indicated times. PH-miniSOG is under the control of *unc-17β* promoter. The *sod-1* mutant does not enhance the Unc phenotype of PH-miniSOG transgenic animals after blue light illumination.

Figure S5 PH-miniSOG efficiently kills GABAergic motor neurons and touch neurons after blue light illumination.

c. Pan-neuronal morphology (labeled by P*rgef-1*-mCherry) in P*unc-25*-PH-miniSOG transgenic animals before and 24h after 4 min blue light illumination. Note that PH-miniSOG activation kills motor neurons but not other nearby neurons. White arrows indicate motor neurons. Scale, 10 μm.

d. PH-miniSOG expression in touch neurons caused ALM, AVM, PLM, and PVM cell degeneration 24 h after 4 min blue light illumination. White arrows indicate degenerated cells. Scale, 10 μm.

Figure S6. The Q103L miniSOG variant enhances cell ablation efficacy

a. Animals expressing PH-miniSOG or PH-miniSOG (Q103L) in the epidermis were indistinguishable from wild type before blue light illumination. Quantitation of locomotion velocity of PH-miniSOG, PH-miniSOG (Q103L) transgenic and WT animals, using the multi worm tracker. Numbers are the animals that were analyzed.

b. Onset of paralysis in animals expressing epidermal PH-miniSOG or PH-miniSOG (Q103L), after 30 sec 2 Hz blue light illumination. Numbers are the animals that were analyzed in three independent experiments.

c. Animals expressing PH-miniSOG (Q103L) in the cholinergic motor neurons are superficially wild type prior to blue light illumination. Quantitation of locomotion velocity of P*unc-17β*-PH-miniSOG (Q103L) transgenic and N2 (WT) animals using the multi worm tracker. Numbers are the animals that were analyzed.

d. Onset of paralysis in animals expressing cholinergic PH-miniSOG and PH-miniSOG (Q103L) animals after 20 sec continuous blue light illumination. Numbers are the animals that were analyzed in three independent experiments.

Supplemental Movie Legends

Movie S1. P*col-19*-miniSOG. With and without blue light illumination. These transgenic animals display normal locomotion before and after illumination. Time: min.

Movie S2. P*col-19*-mito-miniSOG. With and without blue light illumination. These transgenic animals display normal locomotion before illumination and are paralyzed immediately after 10 min blue light (2 Hz) illumination. Time: min.

Movie S3. P*col-19*-PH-miniSOG. With and without blue light illumination. These transgenic animals display normal locomotion before illumination and are paralysed immediately after 2 min blue light (2 Hz) illumination. Time: min.

Movie S4. P*col-19*-myr-miniSOG. With and without blue light illumination. These transgenic animals display normal locomotion before illumination and are paralyzed immediately after 4 min blue light (2 Hz) illumination. Time: min.

Movie S5. P*unc-17β*-PH-miniSOG. With and without blue light illumination. These transgenic animals display normal locomotion before illumination and are Unc immediately after 3 min blue light (2 Hz) illumination. Time: min.

Movie S6. P*unc-25*-PH-miniSOG. With and without blue light illumination. These transgenic animals display normal locomotion and are Unc immediately after 4 mins blue light illumination. Time: min.

Movie S7. P*myo-3*-PH-miniSOG. With and without blue light illumination. These transgenic animals display normal locomotion before illumination and are paralyzed immediately after 4 min blue light (2 Hz) illumination. Time: min.

References:

1 Qi, Y. B., Garren, E. J., Shu, X., Tsien, R. Y. & Jin, Y. Photo-inducible cell ablation in *Caenorhabditis elegans* using the genetically encoded singlet oxygen generating protein miniSOG. *Proc Natl Acad Sci U S A* **109**, 7499-7504 (2012).
